# TRACER: a resource to study the regulatory architecture of the mouse genome

**DOI:** 10.1186/1471-2164-14-215

**Published:** 2013-04-02

**Authors:** Chao-Kung Chen, Orsolya Symmons, Veli Vural Uslu, Taro Tsujimura, Sandra Ruf, Damian Smedley, François Spitz

**Affiliations:** 1 European Bioinformatics Institute - European Molecular Biology Laboratory, Wellcome Trust Genome Campus, Hinxton, Cambridge CB10 1SD, UK; 2 Developmental Biology Unit - European Molecular Biology Laboratory, Heidelberg, Germany; 3 Present address: Welcome Trust Sanger Institute, Wellcome Trust Genome Campus, Hinxton, Cambridge, CB10 1SD, UK

**Keywords:** Gene regulation and expression, Genome organisation, Regulatory landscapes, Chromosomal engineering, Mouse models of human structural variation

## Abstract

**Background:**

Mammalian genes are regulated through the action of multiple regulatory elements, often distributed across large regions. The mechanisms that control the integration of these diverse inputs into specific gene expression patterns are still poorly understood. New approaches enabling the dissection of these mechanisms *in vivo* are needed.

**Results:**

Here, we describe TRACER (http://tracerdatabase.embl.de), a resource that centralizes information from a large on-going functional exploration of the mouse genome with different transposon-associated regulatory sensors. Hundreds of insertions have been mapped to specific genomic positions, and their corresponding regulatory potential has been documented by analysis of the expression of the reporter sensor gene in mouse embryos. The data can be easily accessed and provides information on the regulatory activities present in a large number of genomic regions, notably in gene-poor intervals that have been associated with human diseases.

**Conclusions:**

TRACER data enables comparisons with the expression pattern of neighbouring genes, activity of surrounding regulatory elements or with other genomic features, revealing the underlying regulatory architecture of these loci. TRACER mouse lines can also be requested for *in vivo* transposition and chromosomal engineering, to analyse further regions of interest.

## Background

**Figure 1:**
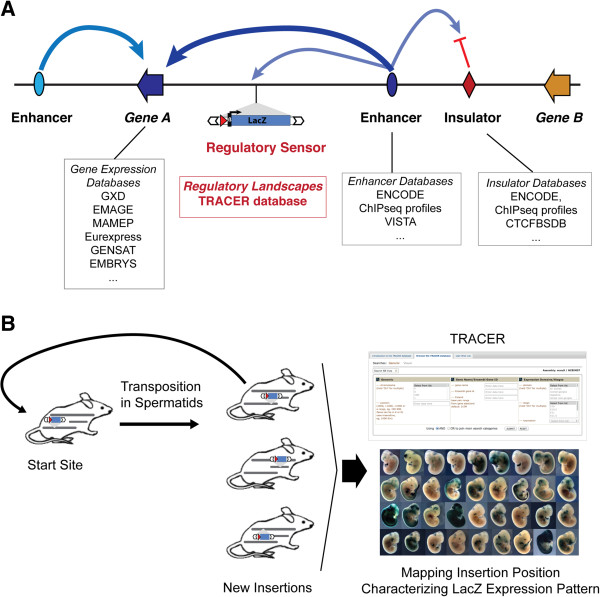
**Genome organisation and TRACER.** (**A**) Schematic representation of a genomic locus, with the different elements that control gene expression, and the specialized databases dedicated to their description (see Websites). The TRACER database displays information from a regulatory sensor that detects the regulatory influences active at its insertion site, outlining the characteristics and extent of genomic regulatory landscapes. (**B**) The GROMIT strategy. A transposon that contains the regulatory sensor can be remobilised *in vivo* from its start site by a transposase transgene (not shown) active only in the male germline [2]. The genomic position of the new insertions and the associated expression pattern defined by LacZ staining in mouse embryos is displayed in the TRACER database.

Genes occupy only a small fraction of mammalian genomes. Accordingly, intergenic regions can extend up to several megabases, and the functional importance of these regions is being growingly recognized [1] (Figure 1). Notably, these regions comprise important elements that control gene expression [3]. Enhancer elements are frequently found hundreds of kilobases away from the promoter of the gene they control, sometimes even separated from it by unrelated genes [4-11]. These remote enhancers can be essential for gene expression, as shown by human disorders resulting from their mutation or disruption by chromosomal rearrangements [12-16]. The importance of these intergenic regions in human phenotypic diversity and disease susceptibility is further emphasized by the significant proportion of risk alleles that have been identified in gene-desert intervals [3,17-20]. Thus, there is a pressing need to better characterize the nature of the regulatory activities embedded in such regions and to obtain animal models to help dissect *in vivo* how variations in these regions contribute to human phenotypes.

Recent progress in whole genome chromatin profiling has led to the identification of chromatin features that are strongly correlated with gene regulatory elements [21-26], opening ways to obtain a comprehensive catalogue of these elements, and a better annotation of the regulatory genome [27]. Databases that document the *in vivo* activities of experimentally validated regulatory elements – mostly enhancers – further complement these approaches [28]. Such datasets on regulatory activity can be compared to gene expression data in developing mouse embryos [29-35]. However one cannot reduce gene expression to a catalogue of the many potential regulatory elements present in the genome (from few hundred thousands to millions [22]). It is equally important to understand the interplay between the different elements present at a locus and how their different inputs are integrated and conveyed to target gene(s). Yet, compared to enhancers, other *cis*-regulatory elements such as silencers are much more elusive, despite their essential role in gene expression. Similarly important are the mechanisms that define the range and specificity of enhancer-promoter interactions. Indeed, changes in the relative position of genes and regulatory elements by chromosomal rearrangements and structural variations can alter gene expression with dramatic consequences [36-40]. Understanding these situations and the associated mechanisms requires approaches that complement the available catalogues of elements and provide a functional integrated view of the genome regulatory architecture.

For this purpose, we have developed an approach based on the distribution of a regulatory sensor gene throughout the mouse genome [2] (Figure 1B). The regulatory sensor consists of a LacZ reporter gene, which is driven by a minimal promoter that has no specific activity on its own but responds faithfully to endogenous enhancers. This regulatory sensor therefore uncovers the *regulatory potential* associated with a given genomic position, which results from the collective action of the different regulatory elements that act on this position. It thus reveals, in an operational manner, the gene regulatory activities within poorly characterized regions, or where annotation for activity is indirect (eg. chromatin profiling) or out of the proper genomic context (eg. transgenic assays). Importantly, the minimal promoter used does not display any obvious tissue- or enhancer-type bias, and the observed expression patterns often overlap with the ones of neighbouring genes [2]. The basic principle of the strategy is analogous to an enhancer-trap [41]; however, the sensor used in our approach has minimal impact on endogenous gene expression [2] and therefore reveals regulatory activities without titrating them away from their natural target genes.

This regulatory sensor is carried in a *Sleeping Beauty* transposon, which can be distributed randomly in the mouse genome, by remobilisation in the male germline [2]. Owing to the efficiency of this *in vivo* transposition system, we have recovered, identified and characterized a large number of insertions that provide a direct view of the regulatory activities associated with specific genomic regions. Furthermore, as the transposons used also carry a *loxP* site, the different lines can be used for *in vivo* chromosomal engineering, to generate mice with targeted deletions or duplications, or segmental aneuploidies [2,42-44]. The local hopping behaviour of *Sleeping Beauty* makes each line a potential starting point to scan a region of interest [45]: with our germline-specific transposase transgene, the remobilization rate ranges from 10 to 45%, depending on the starting site, and more than 15% of new insertions are within 1 Mb of the starting point. Thus, a research group with access to a limited number of cages can nonetheless set up a regional screen for its region of interest.

To provide a simple and useful access to the expression patterns and the mouse insertion strains generated with this on-going project, we have designed the **T**ransposon- and **R**ecombinase-**A**ssociated **C**hromosomal **E**ngineering **R**esource (TRACER) database. This new database is freely accessible at http://tracerdatabase.embl.de/. It constitutes a substantial improvement over the previous one that was established to display the data from a limited pilot screen [2]. The new database comprises novel features that allow users to browse and perform refined searches of insertion sites by position and/or expression patterns. The dataset is also now much larger (4-fold increase, with about 1500 insertions in July 2012), and is growing steadily. This web-based database not only provides information on regulatory activities present along the mouse genome but also gives access to a large collection of mice for engineering chromosomal rearrangements in non-genic intervals.

## Construction and content

### Dataset

**Figure 2:**
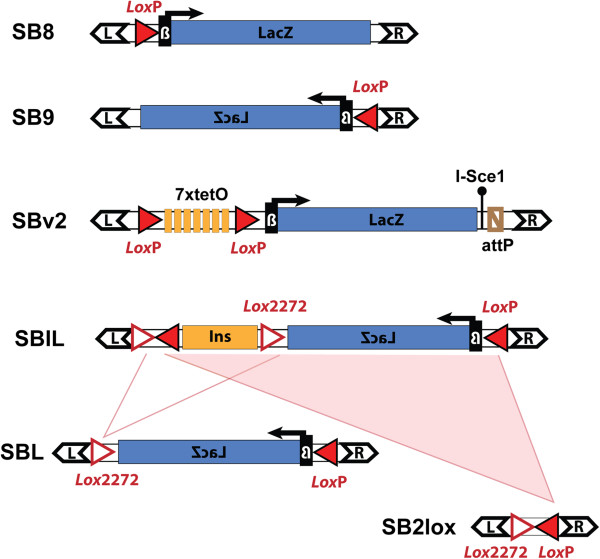
**The different transposons used in TRACER.** Within the left and right inverted/direct repeats of the SB transposon (black double arrows) different cargoes have been cloned that can be utilised for various purposes. Most lines contain an insertion of either the SB9 or SB8 transposon, comprising a LacZ reporter gene with a SV40 polyA sequence (blue rectangle), driven by a synthetic promoter composed of 50 bp of the human beta-globin promoter (black box with β), and a *loxP* site (red triangle). Newer transposons with additional features have been constructed, and mice with these transposons are being produced. Additional modules comprise sites for the PhiC31 integrase (attB), and the I-Sce1 meganuclease, which open possibilities to use the transposon as a docking site for incoming cassettes [46-48]. TetO binding sites can be used to recruit fusion proteins [49]. SBIL contains an insulator/enhancer-blocker element (Ins, orange block, from the chicken HSS4 element [50]) flanked by *loxP* and *lox2272* (white triangle with red contour). Cre-mediated recombination can transform this transposon into different derivatives (SBL or SB2lox), depending on which *lox* sites are used.

In July 2012, the TRACER database contained information on 1467 insertions, 643 of which had been characterized for expression in mouse embryos (mostly at stage E11.5). Specific expression patterns were reported for 344 insertions, documented and annotated by 852 pictures. The dataset is updated regularly, with new insertions and new expression data. Most insertions were obtained with SB9 or SB8 transposons, which contain the regulatory sensor and a *loxP* site cloned in one or the other orientation in the *Sleeping Beauty* transposon. Newer versions of the transposon with additional features have been developed (Figure 2) and will be introduced in the database when mice with such insertions will be available.

### Methodology and population of database

All our data is stored on a MySQL 5.5.15 RDBMS community server (GPL). Server side programming languages are PHP and PERL CGI. CSS and the javascript framework jQuery render the client data display and graphical user interfaces.

As well as the external user interfaces described below, the TRACER database has internal interfaces restricted to contributing members and requiring login for authentication. These internal interfaces have all the LIMS (laboratory information management system) components required for uploading data, curation of lines and various administration purposes.

The main internal interface allows lab staff to add all the text annotation, and insert sequence and image files associated with a particular TRACER line. There is also a batch upload interface for multiple insert sequences. The backend code automatically cuts the sequence down to just the insert, verifies the mutagen tag is present and the genomic sequence starts with ‘TA’. The batch sequence submission tool is automatically coupled to the UCSC BLAT service with standard parameters (http://genome.ucsc.edu/) to determine the best alignment and genomic location for each insert. When there are multiple good alignments, user intervention is possible to select the best genomic location. An input form is then populated for the aligned sequence along with any existing data for the line. A similar batch upload interface exists for the parsing of the expression image and annotation files. Internal users can also edit annotations for existing lines using a separate curation interface.

Many of the interfaces utilise a controlled vocabulary of terms to populate the drop-down menus, reducing the number of typos in the database and preserving the integrity of the data stored in TRACER. An administrative database exists to edit these controlled vocabularies.

The external interface allows users to register interest in particular lines, or - if the user’s genomic region of interest is not yet covered - to wish for such a line when it becomes available. These requests are captured in the database and matching lines are displayed for the curators so they can contact the requesting researcher. For user-defined regions of interest, new matching lines are automatically searched every week, or when triggered through the curator interface.

## Utility

### Searching the TRACER database

**Figure 3:**
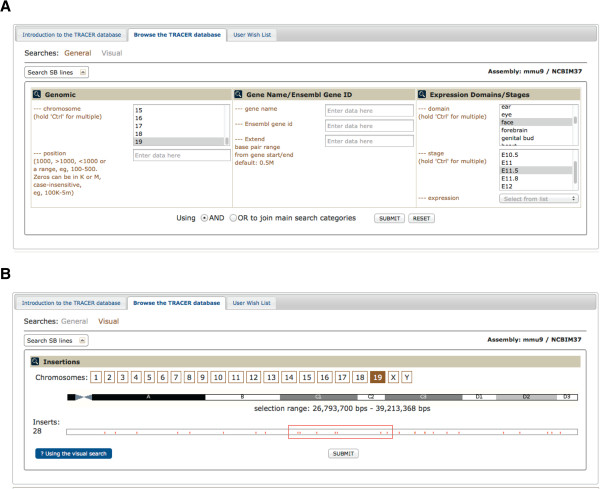
**Searching the TRACER database.** (**A**) TRACER lines can be retrieved (“General search”) either based the genomic position of the insertion as defined by a genomic range or a region around a particular gene, or using the result of the expression assays, with the possibility to specify stage and expression domains of interest. (**B**) Alternatively (“Visual search”), the distribution of insertion sites along a chromosome is visualised, and those within the range defined by a dragable and expandable red rectangle will be returned.

The “Browse the TRACER database” tab takes users to the main search interface of the website (Figure 3A). Insertions of interest can be identified by a variety of options. For genome-centric views, a genomic region of interest can be specified, defined either by chromosomal coordinates (reference genome is MGSCv37/mm9), or by a gene name (“associated gene name” from Ensembl database) and an optional user-defined flanking region (default is 0.5 Mb). In addition to this “General” option, one can perform a “Visual” search by clicking on the link at the top of the search window (Figure 3B). Users can view the distribution of insertions across each chromosome and drag a rectangle to define the region they want to retrieve lines. Searches can also be carried out based on expression patterns, using criteria such as positive/negative, expression domains and expression stages. The label “negative” for expression means that no specific expression patterns was scored for this insertion at the embryonic stages assayed, whereas an insertion is labelled as “positive” if specific expression is detected at least at one stage of development. The majority of the insertions have been assayed at E11.5, but some data is available at other stages (E10 to E13). Expression domains are annotated using a simplified controlled vocabulary (e.g. branchial arches, cranial ganglia, digestive, dorsal root ganglia, ear, eye, face, forebrain, genital bud, heart, hindbrain, limb, midbrain, neural tube, somites, urogenital, others or widespread), which is compatible with the one used by the Vista Enhancer Database [28], in order to facilitate comparison of the two datasets.

### Display and download of data

**Figure 4:**
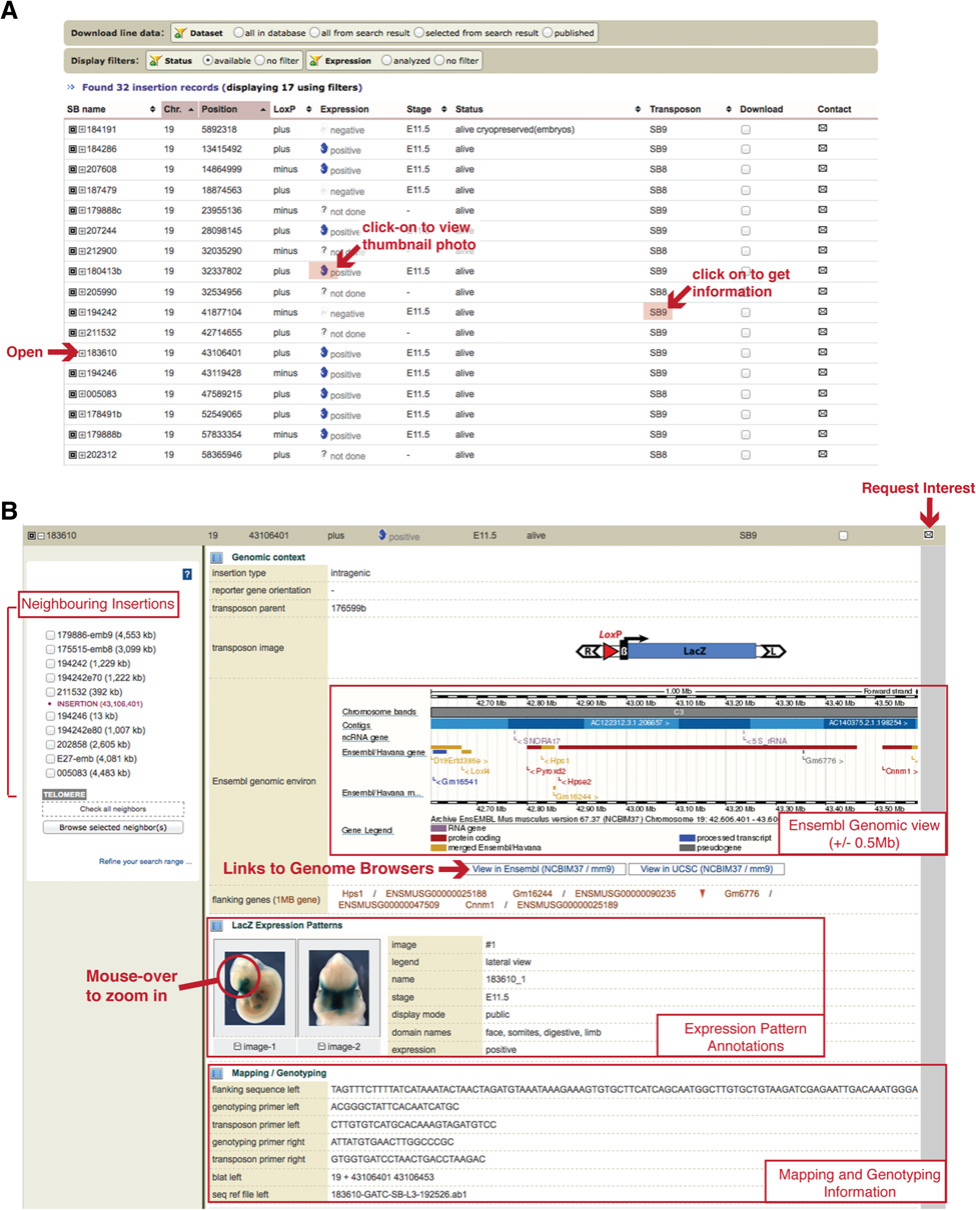
**Presentation of TRACER data.** (**A**) Summary display of results for a TRACER search. For each line the TRACER name, genomic position, orientation of the *loxP* site, summary of expression (expressed/positive, not expressed/negative, not done; developmental stage(s) assayed), status (alive, cryopreserved, not maintained, newly created) and the transposon type are displayed. The final two columns allow users to select the data for download, or to register interest in the line. Clicking on the “open” icon gives access to the detailed information of an insertion of interest. Quick access to a thumbnail photo of a representative embryo and to the corresponding transposon is possible by a simple click on the corresponding zone (**B**) Detailed view of a TRACER mouse line. The top section reveals basic information about the insertion, including the type of insertion, the name of the parent insertion, the orientation of the *loxP* site and a visual representation of the transposon in the correct orientation. The first panel shows the genomic context of the insertion in a snapshot from the Ensembl genome browser (Ensembl Genomic view +/−0.5 Mb) along with links to view the insertion point in the Ensembl or UCSC genome browsers. The second panel shows photos and annotations of the expression patterns of the regulatory sensor. The images can be mouse-overed to reveal a high-resolution zoomed-in view, and the annotation of expression domains is displayed. The third panel shows information related to mapping and genotyping, including the sequence obtained from the mapping procedure, and the sequences of the primers used to genotype animals carrying this specific insertion. The interface in the panel on the left allows neighbouring insertions to be selected for detailed analysis of a region’s regulatory potential.

Results are returned as a table (Figure 4A) with one row per insertion and sortable columns displaying:

•The internal identifier of the mouse line in the TRACER database.

•The genomic position of the insertion (chr/position ; based on MGSCv37/mm9 genome assembly).

•The orientation of the *loxP* site in the transposon. “Plus” corresponds to the following orientation: centromere – 5^′^-ATAACTTCGTATA**GCATACAT**TATACGAAGTTAT- 3^′^ telomere. For comparison, *loxP* sites targeted by the International Knockout Mouse Consortium in genes transcribed from the plus strand (http://www.knockoutmouse.org/about/targeting-strategies) have the same orientation than TRACER “plus” *loxP*. Depending on the specific transposon, the orientation of the other features (transposon ends, reporter gene) varies: they are indicated and represented in the expanded view available by clicking on the “expand” icon.

•An icon and text, indicating whether expression analysis has been performed and whether LacZ reporter expression has been detected. The developmental stage(s) for which information is available are indicated in the next column. Expression assay is “positive” if the insertion showed LacZ staining at least at one of the stages assessed.

•The status of the insertion, indicating whether animals carrying the insertion are available. Insertions that were identified in F0 embryos, that couldn’t be established from the founder or were discontinued, are labelled as “not maintained”. Insertions “available” for further use or analysis fall under three categories: “alive” (line established with mice available in small numbers), “cryopreserved” (either as embryos or sperm) and “new” (usually corresponding to a new insertion, with only the founder animal). The status of an insertion is dynamic: not all “new” insertions are established, and depending on circumstances, “alive” ones may become “cryopreserved” or “not maintained”.

•Transposon type: most of the available lines harbour a simple regulatory sensor with a lacZ reporter and a single *loxP* site, in one or the other orientation relative to the transposon ends (SB8 and SB9). New transposons with additional features have been constructed (see Figure 2), and lines containing them are being established and will be added to the resource. Detailed maps and sequences of available transposons are available on the Tracer website.

The final two columns display a checkbox to download the complete set of information available for an insertion, and an email link to indicate interest in a specific insertion. The toolbar buttons above the results table can be used to filter the search results, and to show only available lines and/or lines with expression data.

Further details on a given insertion can be seen by clicking the expand icon next to each record (Figure 4B). The first section describes the genomic context of the insertion. It lists whether the insertion is located in a gene desert (a gene-free region larger than 500 kb), intergenic (less than 500 kb-long), intronic or exonic region, specifies the orientation of the reporter gene, and the parental insertion line from which the insertion was obtained. This section also contains a schematic of the transposon construct, the genomic environment and flanking genes in a snapshot from the Ensembl genome browser [51] along with links to view the insertion point in Ensembl or the UCSC genome browser [52].

The second section shows the LacZ expression patterns obtained for the insertion, when available. Mousing over each thumbnail image show a zoomed-in, trackable high-resolution view of the image. In addition, the stage and viewpoint of the image is recorded along with annotations using the expression domain categories detailed above. One can switch from one image to another one by clicking on the corresponding thumbnail.

The final section shows details regarding how the genomic position of the insertion was determined, such as the flanking sequence(s) obtained (trimmed to the TA dinucleotide duplicated upon Sleeping Beauty insertion [53]), and where this sequence mapped where this sequence mapped to genome using BLAT [54]. When available, primers that have been used to genotype embryos and mice for this specific insertion are indicated.

The left hand panel of the expanded section contains an interface that displays lines with insertion points within 5 Mb (or a user-selected range) (Figure 4B). Users can select one or more of these lines, and open a new tab displaying these flanking lines. This feature is particularly useful to compare regulatory activities across large regions, and to delineate the extent of regulatory domains.

Finally, the toolbar below the search interface allows data to be downloaded for the whole TRACER database, the search results, user selected lines or just the lines described in publications referring to the dataset. Additionally, all available images can be downloaded. Requests for higher resolution photos and other questions can be sent to gromit@embl.de. Most LacZ stained embryos has been archived, albeit in limited numbers for each insertion, and may be made available upon request.

### User wish list

**Figure 5:**
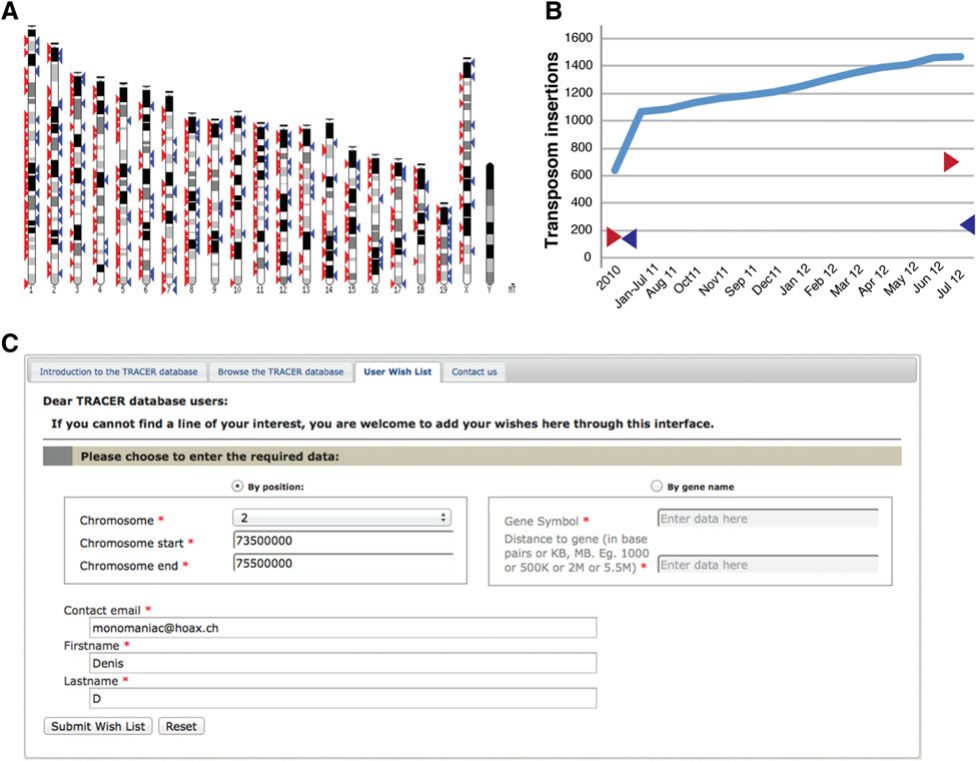
**Evolution of TRACER dataset.** (**A**) Genomic distribution of the insertions with expression annotations (red) and available as lines (blue) (generated with the Karyoview tool from Ensembl – status in July 2012). Insertions on the Y chromosome are not shown as their positions are ambiguous (**B**) The number of mapped insertions increases at a regular pace (light blue line), almost paralleled by expression reports (red cursor). A smaller proportion of these are kept alive or archived by cryopreservation (blue cursor). (**C**) The user wish list allows researchers to register interest for a genomic interval. It ensures that mice with new insertions in this region will be preserved for the scientist who indicated this request. The interval of interest can be defined by position or by gene name.

Although the TRACER database already covers a substantial proportion of the genome, it is likely that individual researchers will be interested to get information and mice with transposons in regions where we haven’t yet identified an insertion. Given the high efficiency of transposition, the number of new insertions identified in on-going remobilisation efforts (~ 10 per week) exceeds our current capacity to keep, expand and cryopreserve all of them (Figure 5). The “User wish list” tab allows scientists to indicate particular genomic regions they are interested in, along with their contact details (Figure 5C). Once an insertion in this region is identified, it is “flagged” for the producing group, so that the corresponding animal is kept, and the interested group will be contacted.

## Discussion

### A functional view of the genome with TRACER

The introduction of a “regulatory sensor” in the genome provides a direct operational readout of the activities that can contribute to gene expression, which surround the insertion point. Similar enhancer-trap screens have widely been used in *Drosophila*[41] and to some extent in zebrafish [55-58], providing information about genes and genomes, as well as a series of useful markers and tools. Their use in mice has been limited [59,60], in part due to the low throughput of transgenesis, and technical difficulties of generating single-copy insertions. The development of robust and efficient *in vivo* transposition systems [2,61-63], as shown here, or the use of lentiviral transgenesis, as recently described elsewhere [64], open new exciting possibilities to conduct such screens in an efficient and affordable manner.

**Figure 6:**
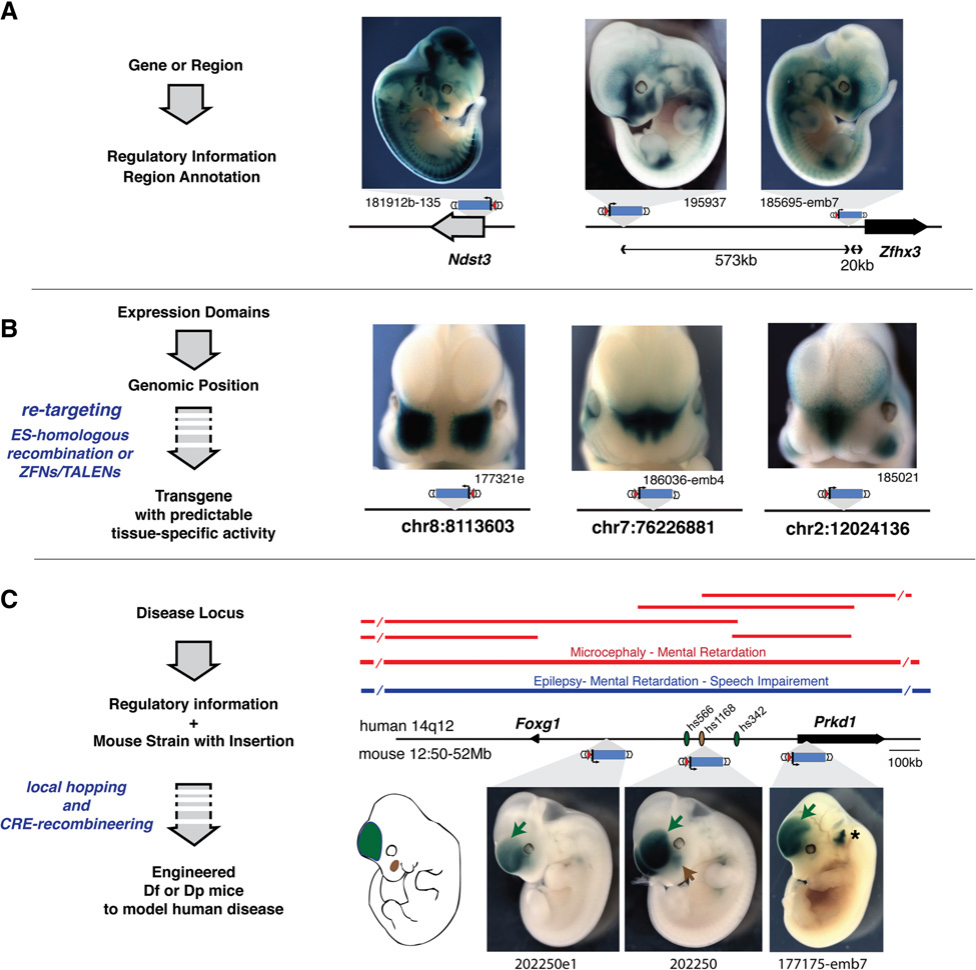
**Examples of potential applications of TRACER.** (**A**) Using TRACER to obtain information about the regulatory potential of a specific interval. Two loci (*Ndst3* and *Zfhx3*) are given as an example, with photos of lacZ-stained embryos with insertions close or far from these genes. (**B**) Searching TRACER for insertions with specific expression patterns can identify genomic positions where an inserted transgene will display a predicted activity. Three insertions with different patterns of expression in the fronto-nasal process are shown as examples. Such specific patterns can subsequently be exploited to re-target other transgenes with expression in the predicted domains through different techniques. (**C**) Overlapping deletions (red bars) and duplications (blue bars) over 14q12 entered in the DECIPHER database (http://decipher.sanger.ac.uk/) are associated with similar clinical phenotypes. TRACER insertions in the orthologous interval show that it corresponds to a large regulatory landscape associated with forebrain (green) and facial (brown) expression domains in E11.5 (SB-202250e1 and SB-202250) and E12.5 (SB-177175emb7) mouse embryos. LacZ expression in the ear of embryo SB-177175emb7 (star) is due to the presence of an additional insertion, SBlacC [2]. Enhancer regions (ovals, color-coded depending on their pattern of activity as shown in the schematized embryo; data from VISTA enhancer browser [28]) present in the proximity may contribute to these activities. By local remobilisation and Cre-mediated recombineering, the mouse line SB-202250 may be used to produce mouse models of the human aneuploidies associated with this largely non-coding interval.

Collections of insertions generated by these approaches can provide useful information and tools, and the TRACER database represents a substantial step to capitalise on such a collection, by centralizing and giving access to data and to mouse lines. We present and discuss here briefly some of the possible uses of this database and of the information therein (Figure 6).

By querying the database for a gene or a region of interest, one can identify expression patterns and regulatory activities associated with that location and its surroundings. The observed activity may indicate possible developmental or tissue-specific regulation of genes, and shed light on their physiological roles *in vivo* (Figure 6A). However, we wish to emphasize that the regulatory sensor sometimes reflects only a subset of the expression domains of a given gene [2]. Although the sensor responds accurately to influences from long-range remote enhancers, it is less likely to capture the input of promoter elements that have a limited range of action: tissue-restricted expression of the sensor may therefore represent a tissue-specific modulation of an otherwise broadly expressed gene; yet, this modulation may correspond to important biological functions.

Also, the expression pattern associated with an insertion does not necessarily imply that a corresponding enhancer lies nearby, as illustrated by the shared expression of distant insertions (Figure 6A,C; other examples in [2]). Instead, the sensor reports the collective input at a given position of both positive and negative regulatory elements. Accordingly, comparing the expression pattern of neighbouring insertions to each other and to known enhancer activities [21,65] can reveal important regulatory features. These include the range of action of enhancers, the boundaries of expression domains, the presence of silencers or other repressive or insulating elements that modulate enhancer activity and cannot be obtained from other types of datasets and approaches. In essence, TRACER provides an operational view of the regulatory structure of the mammalian genome, and delineates the extent of the large *regulatory landscapes*[6] that subdivide the genome into functional units. It constitutes a functional counterpart to views obtained by different methods; including, for example, *Genome Regulatory Blocks* that are delineated by the density of conserved non-coding elements and synteny conservation [66,67], *Topological Associated Domains* defined by chromosomal interaction biases [68,69], and *Enhancer-Promoter Units* that are revealed by clusters of coincident promoter-enhancer chromatin signatures [70].

The data present in TRACER identifies genomic positions where an inserted transgene will adopt a highly specific expression profile (Figure 6B). Transgenes that drive the expression of markers to label specific cells (such as fluorescent markers) or of effector genes (for example Cre recombinase) in defined cell-types or embryonic tissues have proven very useful to dissect biological and genetic processes. “Position-effects” (the action of endogenous regulatory elements on transgenes) are usually considered as a problem for transgenic experiments because they lead to partially unpredictable outcomes. With the information displayed in TRACER, one can instead exploit position effects, and select genomic sites that will convey an expression pattern of interest. Importantly, many of these sites are located far from genes, implying that their use would have less functional impact than a gene knock-in. The sensor integrates the inputs of both enhancers and silencers that are acting at its position: consequently, the observed pattern is often more restricted than the one driven by enhancer-only constructs or displayed by the neighbouring genes [2]. Hence, retargeting positions identified in TRACER with a transgene of interest should provide a reliable method to create new tissue- and cell-type specific transgenes. This can be done by homologous recombination in mouse ES cells, but the rapid development of Zinc-Finger or TALE Nuclease-associated targeted transgenesis may offer more efficient alternatives [46,71,72].

In addition to maps of genomic “regulatory landscapes”, TRACER provides access to a large and growing collection of mice with different transposon insertions (around 200 in July 2012). Only few insertions are likely to disrupt genes or key/highly conserved regulatory elements directly. Instead, these mice can be used for other purposes, and in particular for engineering aneuploidies and structural variants. Chromosomal aneuploidies are often found in patients suffering developmental malformations and/or neuropsychiatric disorders. In some cases, single gene-knockout can reproduce the phenotypes observed in human patients; however, for numerous other conditions, such as contiguous gene diseases, chromosomal duplications or rearrangements in non-coding intervals, gene-based alleles do not provide accurate models. Because *Sleeping Beauty* transposons frequently re-insert in the vicinity of their initial position, it is possible to use one insertion in a region of interest to generate additional local re-insertions. These insertions can be (re)combined owing to the associated *loxP* sites, to produce a series of rearrangements of this locus that model genomic alterations found in human patients, and help determine the causal elements or genes (Figure 6C). Such a use of the TRACER resource and GROMIT strategy can be particularly well suited for large gene clusters (eg. proto-cadherins, KRAB-zinc finger genes, olfactory receptors) or gene-deserts associated with human pathologies, complementing the gene-centric resource provided by the International Knockout Mouse Consortium. Given the growing recognition of the biological importance of genomic structural variants for human diseases, we anticipate that TRACER will be a useful resource to rapidly engineer allelic series of structural variants in mouse orthologous intervals, helping to create novel models of human genomic disorders.

## Conclusion

### TRACER database and community

Owing to the dynamic nature of transposon elements, the resource present in TRACER will expand steadily with the number of users. Each lab using this transposon technology to investigate a region of interest by “local” hopping will produce a substantial number of by-products (~ 80% of the new insertions). Even if these insertions may not be useful for the producing lab, they can be of interest for others. TRACER is designed to serve as a central “virtual” repository to share those mice. Further information, including references, detailed maps and sequences of the different transposons and transgenes in use, and protocols for mapping of new insertions are available through the pages of the TRACER website.

To facilitate exchanges, the TRACER database incorporates several features and internal interfaces for contributing groups (automated insertion mapping, annotation and administration). In particular, the “User wish list” feature offers a simple manner to readily “tag” newly generated mice of interest without a major investment or commitment of the producing labs.

## Availability and requirements

The database is accessible at the web addresses:

http://tracerdatabase.embl.de

http://www.ebi.ac.uk/panda-srv/tracer/index.php

### Websites – links

ENSEMBL: http://www.ensembl.org/Mus_musculus/Info/Index

UCSC Genome Browser: http://genome.ucsc.edu/index.html

CTCFBSBD: http://insulatordb.uthsc.edu/

VISTA: http://enhancer.lbl.gov/frnt_page_n.shtml

GXD: http://www.informatics.jax.org/mgihome/GXD/aboutGXD.shtml

EMAGE: http://www.emouseatlas.org/emage/

GENSAT: http://www.gensat.org/index.html

MAMEP: http://mamep.molgen.mpg.de/index.php

EUREXPRESS: http://www.eurexpress.org/

EMBRYS: http://embrys.jp/embrys/html/MainMenu.html

DECIPHER: http://decipher.sanger.ac.uk/

## Competing interests

The authors declare that they have no competing interest.

## Authors’ contributions

CKC, DS and FS designed the database with critical input from OS, VVU, TT, SR. CKC wrote the code, the different interfaces and tools associated with TRACER. OS, VVU, TT, SR and FS provided all data present in the database. DS and FS wrote the manuscript, with input and suggestions from all the other authors. All authors read and approved the final manuscript.
